# Exploring the sonodynamic effects of bacteriochlorophyll a

**DOI:** 10.3389/fbioe.2023.1186897

**Published:** 2023-05-11

**Authors:** Lanqi Jia, Longhao Wang, Yiqiong Song, Xin Pang, Jie Zhao

**Affiliations:** ^1^ Internet Medical and System Applications of National Engineering Laboratory, The First Affiliated Hospital of Zhengzhou University, Zhengzhou, Henan, China; ^2^ School of Pharmacy, Henan University of Chinese Medicine, Zhengzhou, China; ^3^ Department of Pharmacy, The First Affiliated Hospital of Zhengzhou University, Zhengzhou, China

**Keywords:** bacteriochlorophyll a, sonodynamic therapy, reactive oxygen species, lung cancer, antitumor

## Abstract

**Objective:** The purpose of this study was to investigate whether bacteriochlorophyll a (BCA) could be used as a potential diagnostic factor in near-infrared fluorescence (NIRF) imaging and in mediating sonodynamic antitumor effect.

**Methods:** The UV spectrum and fluorescence spectra of bacteriochlorophyll a were measured. The IVIS Lumina imaging system was used to observe the fluorescence imaging of bacteriochlorophyll a. 9,10-Dimethylanthracene (DMA) reagent was used as a singlet oxygen sensor to detect singlet oxygen produced by bacteriochlorophyll a. LLC cells of mouse lung adenocarcinoma were selected as experimental subjects. Flow cytometry was used to detect the optimal uptake time of bacteriochlorophyll a in LLC cells. A laser confocal microscope was used to observe the binding of bacteriochlorophyll a to cells. The cell survival rate of each experimental group was detected by the CCK-8 method to detect the cytotoxicity of bacteriochlorophyll a. The effect of BCA-mediated sonodynamic therapy (SDT) on tumor cells was detected by the calcein acetoxymethyl ester/propidium iodide (CAM/PI) double staining method. 2,7-Dichlorodihydrofluorescein-diacetate (DCFH-DA) was used as the staining agent to evaluate and analyze intracellular reactive oxygen species (ROS) levels by fluorescence microscopy and flow cytometry (FCM). A confocal laser scanning microscope (CLSM) was used to observe the localization in the organelles of bacteriochlorophyll a. The IVIS Lumina imaging system was used to observe the fluorescence imaging of BCA *in vitro*.

**Results:** Bacteriochlorophyll a-mediated SDT significantly increased cytotoxicity to LLC cells compared to other treatments, such as ultrasound (US) only, bacteriochlorophyll a only, and sham therapy. The CLSM observed bacteriochlorophyll a aggregation around the cell membrane and cytoplasm. FCM analysis and fluorescence microscopy showed that bacteriochlorophyll a-mediated SDT in LLC cells significantly inhibited cell growth and caused an obvious increase in intracellular ROS levels, and its fluorescence imaging function suggests that it can be a potential diagnostic factor.

**Conclusion:** The results showed that bacteriochlorophyll a possesses good sonosensitivity and fluorescence imaging function. It can be effectively internalized in LLC cells, and bacteriochlorophyll a-mediated SDT is associated with ROS generation. This suggests that bacteriochlorophyll a can be used as a new type of sound sensitizer, and the bacteriochlorophyll a-mediated sonodynamic effect may be a potential treatment for lung cancer.

## 1 Introduction

Cancer continues to pose a serious threat to global public health because of its high mortality and morbidity (Feng et al., 2019). Lung cancer, which is the most common malignancy and leading cause of cancer-related death worldwide, has the worst prognosis, and most patients were advanced stage or had distant metastasis at the time of diagnosis (Cao et al., 2020). Traditional treatments for lung cancer include surgery, radiation, and chemotherapy, as well as emerging targeted therapy and immunotherapy. In the past decade, considerable progress has been made in therapeutic regimens that combine different treatment approaches (Gong et al., 2017; Tran et al., 2017; Ray et al., 2018). However, these treatments are only effective in some types of cancer and encounter the continuous emergence of drug resistance, strong toxicity and side effects, and poor patient compliance. Therefore, it is important to develop alternative antitumor methods which are safe, effective, and less toxic.

Recently, sonodynamic therapy (SDT) has been considered a promising alternative to conventional therapy. It is also a promising minimally invasive cancer treatment platform (Choi et al., 2020). It is generally believed that the mechanism of sonodynamic therapy is the generation of acoustic cavitation, which is a physical mechanism to produce biochemical phenomena. Ultrasound induces acoustic cavitation, including the formation, growth, and instantaneous rupture of bubbles in the liquid environment (Das et al., 2022). It is a new therapeutic method, which uses low-frequency ultrasound to activate sonosensitizers and then initiates reactive oxygen generation (Yumita et al., 2013). Due to its non-specific mechanism of action and its low ROS mutagenic potential, SDT is effective against almost all cancer cells without resistance. During SDT, the ultrasonic wave is localized, and the sonosensitizers have no inhibition effect. As a result, the risk of systemic toxicity, which is often a problem for conventional therapies, is significantly reduced (Jiang et al., 2018). Another advantage of ultrasound is that it has no invasiveness and superior tissue penetration. SDT has been proven to be highly effective against deep-seated tumors (such as lung cancer), which has greatly exceeded the penetration limits of other new alternatives, such as photo-/hyperthermia (Pan et al., 2018). More importantly, the fact that the majority of sonosensitizers have inherent optical properties makes them useful as imaging probes for the optical diagnosis of cancer (Sennoga et al., 2017). SDT, which serves as a treatment platform, offers an excellent opportunity to improve treatment outcomes and optimize the current treatment of lung cancer by integrating these therapeutic and diagnostic functions into one system (Tachibana et al., 2008).

Safe and efficient sonosensitizers are the most important key to successful cancer SDT. In the current stage, the commonly used sonosensitizers are porphyrins, phthalocyanines, and phenothiazines and some natural products, such as curcumin, hypocrellin B, and hypericin (Costley et al., 2015). Most of these substances have an unstable composition ratio, and their maximum absorptions are in the UV-vis light region (Fan et al., 2016). This may induce skin sensitivity and potential phototoxicity (Abrahamse and Hamblin, 2016). In addition, when the imaging probe is used *in vivo*, this kind of sonosensitizer often faces high optical interference from the intrinsic chromophores of living subjects, which is a major obstacle to their potential application in the field of clinical optics.

Nature always offers rich answers to scientific challenges. Inspired by the photosynthesis of some bacteria in natural environments, bacteriochlorophylls attracted our attention. As the important substances for bacterial photosynthesis (Pfennig, 1967), bacteriochlorophylls can efficiently capture sunlight and transfer the harvested light energy with an energy-conversion efficiency as high as 95% or more, and therefore, they are exploited as potent photosensitizers for photodynamic therapy. More importantly, these bacteriochlorophylls always operate in the near-infrared (NIR, 700–900 nm) spectrum, effectively overcoming phototoxicity and biological tissue absorption and scattering (Schuitmaker et al., 1998). Considering that most sonosensitizers are derived from photosensitizers, and bacteriochlorophylls possess a porphyrin structure, bacteriochlorophylls are potential sonosensitizers for SDT, but to date, the first study is still awaited. Here, bacteriochlorophyll a (BCA) was selected as a model drug and then evaluated for sonodynamic activity. Upon ultrasound irradiation, BCA could effectively produce ROS, providing an excellent *in vitro* inhibition effect on lung cancer cells. For traditional imaging, such as CQDs mentioned by Das, its photoluminescence makes it widely applicable in the field of nanoscience (Ghosh et al., 2022). When it is combined with drugs as nanocarriers, drugs have the functions of fluorescence imaging and *in vivo* labeling. The BCA used in our study is capable of fluorescence imaging with a single drug. Further coupled with inherent fluorescence imaging capacity, the BCA may serve as a promising treatment platform for optical visualization and sonodynamic elimination of cancer. In this study, we used ultrasound to activate BCA for the first time to observe the inhibitory effect of BCA combined with SDT on the growth of mouse lung cancer cells, to explore whether it can be used as a potential diagnosis and treatment factor of near-infrared fluorescence imaging, and to mediate the antitumor effect of sonodynamic therapy.

## 2 Materials and methods

### 2.1 Sensitizer

Bacteriochlorophyll a was purchased from Frontier Specialty Chemicals Inc. The medicine was stored at 4°C in the dark until further use. A measure of 1 mg of BCA was dissolved in 100 μL of dimethyl sulfoxide (DMSO), and then 900 μL of pure water was added. An ultrasonic crusher was used to dissolve the mixture completely. BCA solution with a concentration of 1 mg/mL was obtained.

### 2.2 Characterization of bacteriochlorophyll a

The UV spectrum and fluorescence spectra of BCA were measured using a full-wavelength enzyme marker, and the scanning range of the enzyme marker was set at 350–900 nm. For NIR-FL (Near Infrared-Fluorescence) imaging, the samples were recorded using an IVIS Lumina imaging system with the Cy 5.5 channel. Different concentrations of BCA (0, 5, 10, 20, 40, and 80 μM) were dissolved in H_2_O and DMSO, respectively. Then, they were resuspended in 2% agarose for imaging evaluation. The generation of singlet oxygen from BCA was studied with 9,10-dimethylanthracene fluorescent reduction. Subsequently, DMA was added to obtain the final concentration of 20 μM. The fluorescence intensity of DMA was measured at an excitation of 360 nm and emission of 380–550 nm.

### 2.3 Cell culture

The mouse lung cancer cell line LLC cells were obtained from the National Collection of Authenticated Cell Cultures, preserved by the Precision Laboratory of the National Telemedicine Center of The First Affiliated Hospital of Zhengzhou University. The cells were grown in high-glucose Dulbecco’s modified Eagle’s medium (DMEM) supplemented with 10% fetal bovine serum (FBS) and 100 U/mL penicillin, streptomycin, and neomycin. The cells were incubated at 37°C and 5% CO_2_ in a humidified incubator. The cells were cultured in a fresh medium every day and passaged every 2 days. Prior to each experiment, logarithmic growth-phase cells in good condition were collected and digested with trypsin digest and stored until further used.

### 2.4 Uptake of BCA by cells

To investigate the effect of co-incubation time on the uptake of BCA by LLC cells, the LLC cells were cultivated on a flat bottom plate (12-well culture plate). A measure of 1 mL of BCA was added to each well and incubated in the incubator for 0, 1, 2, 4, 8, and 12 h, respectively. The cells were then collected, and the optimal uptake time of BCA by LLC cells was detected by flow cytometry.

The cells were incubated with BCA (40 μM) for 2 h at 37°C. Free medicines were washed with phosphate-buffered saline (PBS). The cells were fixed with paraformaldehyde for 10 min; the nuclei were labeled with DAPI (4′,6-diamidino-2-phenylindole) stain for 20 min and then washed with PBS to remove the residual DAPI stain. Finally, an anti-fluorescence quenching agent was added to the cells to avoid light, and the cells were sent for inspection. BCA binding and morphological changes of the LLC cells were observed using a CLSM.

### 2.5 Cytotoxicity and ultrasound treatment

The LLC cells were divided as follows: a blank control group without any treatment, BCA-only group, pure ultrasound only with ultrasonic irradiation treatment group (1.5 W/cm^2^, 1.0 MHZ), and BCA-mediated sonodynamic therapy (SDT) combined with ultrasonic irradiation treatment group (n = 3).

The cytotoxicity of BCA to LLC cells was assessed. The LLC cells were cultivated on a 96-well flat bottom culture plate. BCA dilutions of different concentrations (0, 5, 10, 20, 40, and 80 μM) were used to co-incubate the cells for 2 h. Then, the CCK-8 reagent (10 μL/well) was added, and the cells continued to be incubated in the incubator for 2 h. The absorbance at 450 nm for each group was detected by an multifunctional enzyme marker, and the cytotoxicity was calculated using Eq. 1:

$$Cell\;survival\;rate\;\left(\%\right)=\left(OD\;value\;of\;experimental\;group-OD\;value\;of\;blank\;control\;group\right)/\left(OD\;value\;of\;control\;group-OD\;value\;of\;blank\;control\;group\right)\times 100\%.$$ (1)

The LLC cells were cultivated on a 96-well flat bottom culture plate. The LLC cells were subjected to ultrasonic treatment 2 h after BCA incubation (40 μM). The cell culture plate was placed on the case that was full of ice without the cover before the ultrasonic treatment. A 1-cm-diameter planar transducer emitting radio waves was fixed at the bottom of the box so that the ultrasound beam pointed upward. The distance from the base of the culture plate to the sensor was 1.0 cm. The spatial mean ultrasonic intensity at 1.0 MHz was 1.5 W/cm^2^. All experiments were randomly divided into four groups: BCA-mediated SDT, ultrasound sonication only, BCA treatment only, and sham sonication as a negative control. For BCA-mediated SDT, BCA (40 μM) was used to incubate the cells prior to ultrasound exposure. The cells in the single group of ultrasounds only received ultrasonic treatment and did not receive BCA treatment. Only BCA was used to incubate the cells in the single BCA group without ultrasonic ultrasound. No treatment was given to the cells of the sham sonication group.

### 2.6 Calcein acetoxymethyl ester/propidium iodide (CAM/PI) double staining

The CAM and PI working solutions were diluted with a serum-free medium at a ratio of 1:1000, respectively, to produce a stock solution. The LLC cells were randomly divided into four groups on a flat plate (six-well): BCA-mediated SDT, ultrasound sonication only, BCA treatment only, and sham sonication as a negative control. After the treatment of each group, CAM staining working solution was first added at 1 mL per well to completely cover the adherent cells. The cells were incubated at 37°C in the dark for 30 min and then washed with PBS. Then, PI staining working solution was added at 1 mL per well to completely cover the adherent cells. The cells were incubated at 37°C in the dark for 30 min and washed with PBS. Finally, an anti-fluorescence quenching agent was added and then immediately observed using a fluorescent microscope. The images were recorded using a camera.

### 2.7 Intracellular reactive oxygen species (ROS)

The DCFH-DA and serum-free cell culture medium were diluted at a ratio of 1:1000 to produce a stock solution. The LLC cells were randomly divided into four groups on a flat plate (six-well): BCA-mediated SDT, ultrasound only, BCA therapy, and false sonication. After the treatment of each group, 1 mL DCFH-DA solution was added per well to completely cover the adherent cells. The cells were incubated at 37°C for 20 min without light and then washed with PBS to remove the residual 2,7-dichlorodihydrofluorescein-diacetate (DCFH-DA) stain. Finally, an anti-fluorescence quenching agent was added to the cells to avoid light and sent for inspection. The generation of ROS in the cells of each group was observed under a fluorescence microscope. Intracellular ROS was also analyzed using a flow cytometer with DCFH-DA staining. LLC cells were processed in groups according to the aforementioned method. After loading the probes, the cells were collected and measured by flow cytometry, and the data were analyzed.

### 2.8 Confocal laser scanning microscope

The cells were incubated with BCA (40 μM) for 2 h at 37°C. Free medicines were washed with phosphate-buffered saline. The cells were fixed with paraformaldehyde for 10 min, and the nuclei were labeled with DAPI stain for 20 min and then washed with PBS to remove the residual DAPI stain. Experiments were randomly divided into three groups: lysosomes, endoplasmic reticulum, and mitochondria were stained with Lyso-Tracker Green, ER-Tracker Green, and Mito-Tracker Green, respectively, for 20 min and then washed with PBS to remove probes that are not loaded into cells. Finally, an anti-fluorescence quenching agent was added to avoid light and sent for inspection. BCA binding and morphological changes in the LLC cells were observed using a CLSM.

### 2.9 *In vitro* FL imaging

Different concentrations of BCA (0, 5, 10, 20, 40, and 80 μM) were incubated with LLC cells at 37°C for 2 h. The same concentration of BCA was incubated with LLC cells at 37°C for different time durations (0, 1, 2, 4, 8, and 12 h). The LLC cells were harvested by centrifugation (at 1,800 rpm for 3 min) and then resuspended in 2% agarose for imaging evaluation. An IVIS Lumina imaging system was used to record FL images. Signal strength was assessed through ROI analysis.

## 3 Results and discussion

### 3.1 Characterization of BCA and potential sonodynamic effects of BCA

**FIGURE 1:**
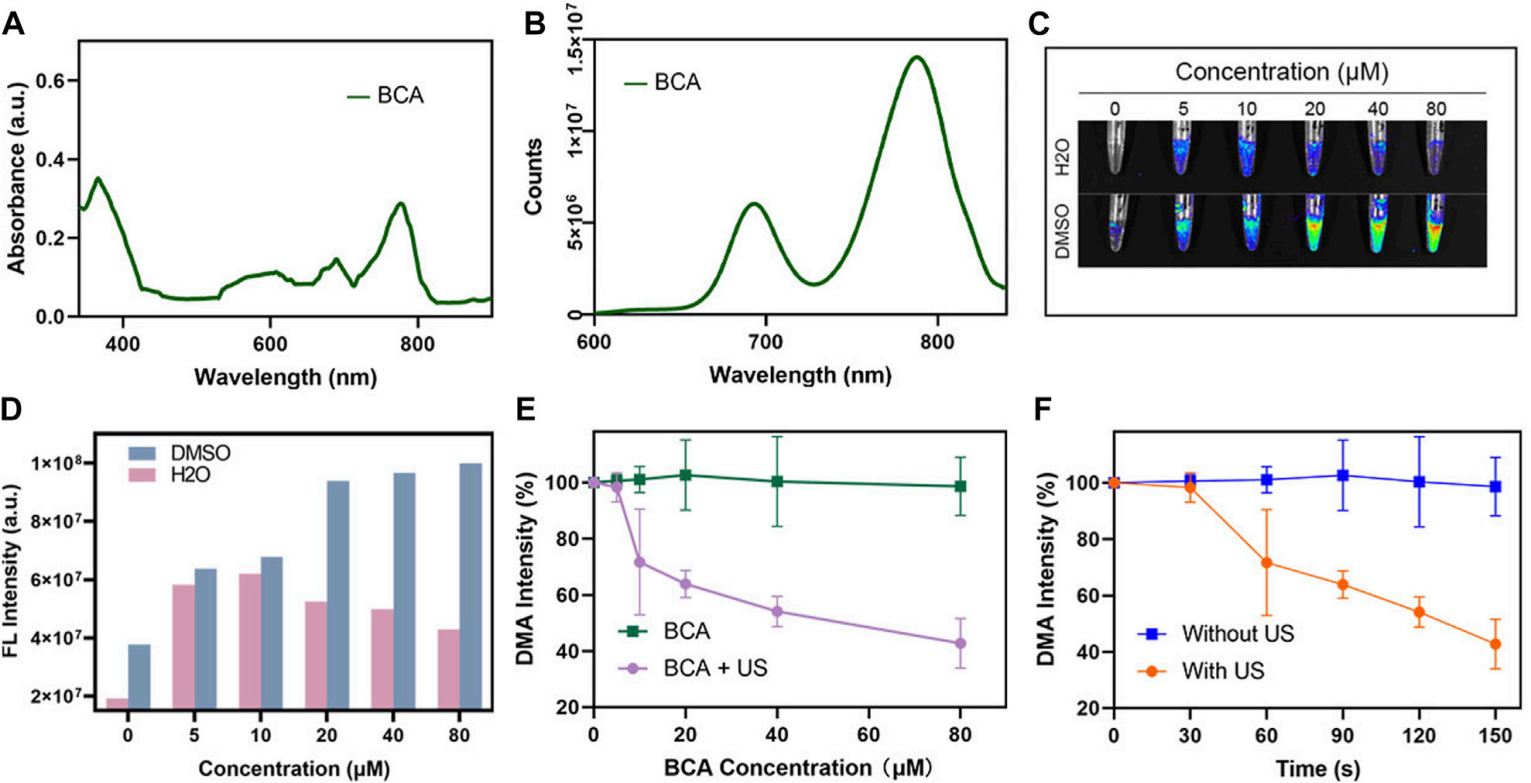
**(A)** UV-vis spectra of BCA in DMSO. **(B)** Fluorescence spectra of BCA in DMSO. **(C)** FL imaging signal changes of different concentrations of BCA dissolved in DMSO and H_2_O. **(D)** Statistic comparison of FL intensity with different BCA concentrations. **(E)** Singlet oxygen generation of BCA upon ultrasound activation. **(F)** Changes in FL intensity of DMA under different conditions (the data are shown as the mean ± SD; *n* = 3 per group).

To evaluate the fluorescence imaging capability of BCA, fluorescence spectra and ultraviolet absorbance of BCA were examined. The UV–NIR spectra of BCA (Figure 1A) showed that the characteristic absorption peaks of BCA were found at approximately 380, 680, and 780 nm. A fluorescence intensity analysis of BCA also confirmed that BCA in the near-infrared region had obvious fluorescence absorption (Figure 1B), which was of great significance for imaging diagnosis in clinics. Then, we evaluated the fluorescence (FL) imaging capability of BCA. As expected, the BCA’s FL signal was gradually enhanced as the BCA concentration increased (Figure 1D), which was clearly visible (Figure 1C). It indicates that the BCA may be a promising contrast agent for fluorescence imaging.

ROS generation is the key factor dominating the efficiency of SDT. The generation of ROS is a key factor in SDT efficiency. Here, we explored whether BCA can be used as an excellent ultrasound-sensitized agent by judging the efficiency of producing reactive oxygen species. Singlet oxygen (^1^O_2_) is thought to be the dominant ROS, which plays an important role in the cytotoxicity of most sonosensitizers. In this study, the production of ROS after US activation of BCA was first assessed using a commercial singlet oxygen (^1^O_2_) probe, 9,10-dimethylanthracene (DMA). There is an irreversible reaction between the detector and ^1^O_2_, which results in a fluorescence quenching effect. Therefore, the high-fluorescent degradation of DMA typically means high ^1^O_2_ generation. As shown in Figures 1E, F, no reduction in fluorescence intensity was observed in samples treated with BCA only, whereas the combination of BCA and US stimuli demonstrated concentration-dependent fluorescence bleaching, suggesting that BCA-mediated SDT effectively generated ^1^O_2_. When further combined with the great FL property, BCA can serve as a potential treatment agent for FL imaging and SDT of tumor.

### 3.2 Uptake of BCA in LLC cells

**FIGURE 2:**
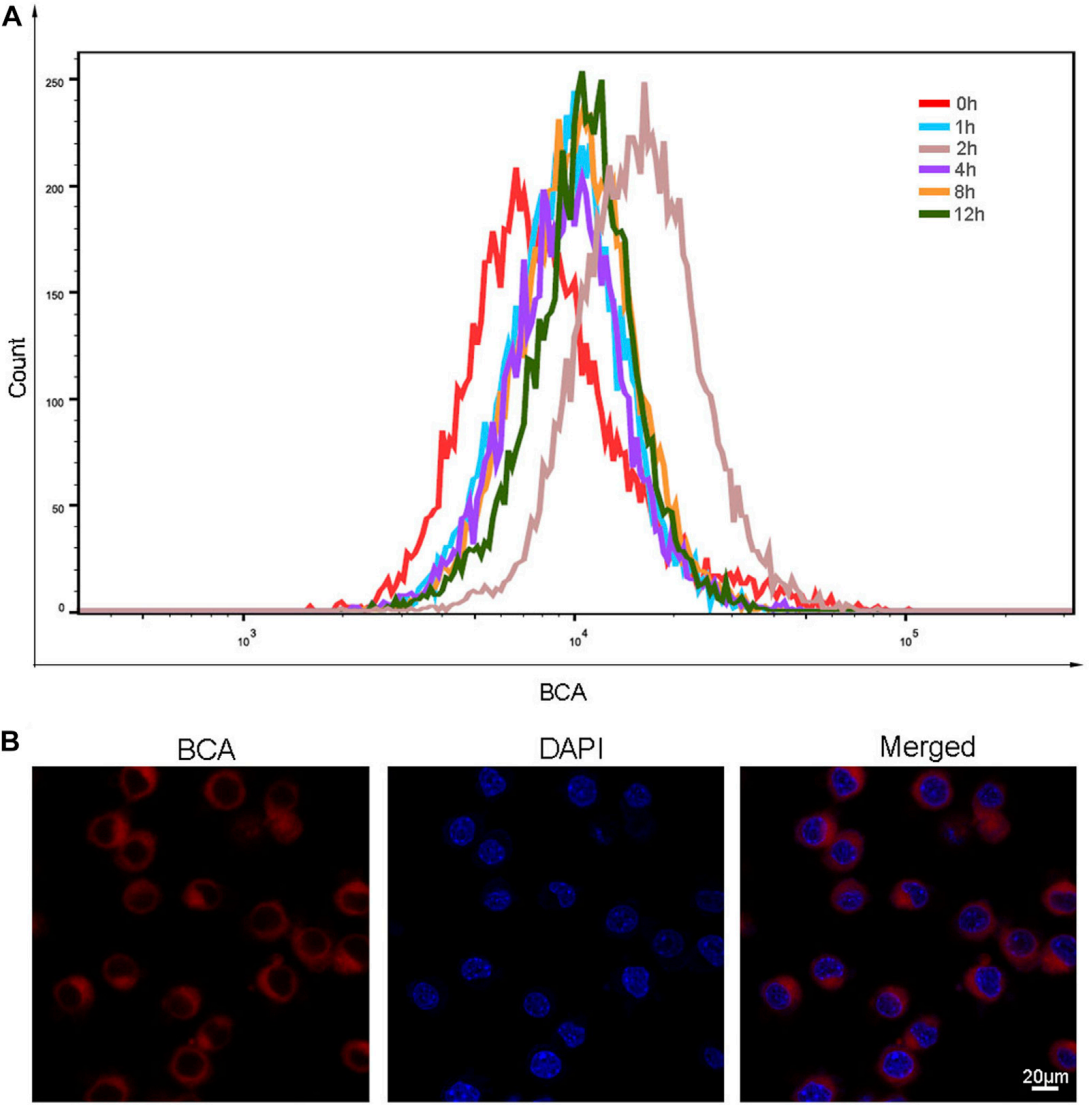
**(A)** Cellular uptake profile of BCA nanoparticles as a function of incubation time. **(B)** Intracellular uptake of BCA in LLC cells, and cell nuclei were counter-stained with DAPI.

After determining the favorable properties of BCA, we then explored whether BCA can be taken up by cells. The treated cells were incubated with the same concentration of BCA for 0, 1,2, 4, 8, and 12 h, and then the cells were collected and detected by FCM. As shown in Figure 2A, when cells were incubated with BCA for 2 h, the cellular uptake of BCA was the highest. As shown in Figure 2B, the red fluorescence provided by BCA distributed around the cell membrane and in the cytoplasm, and the cell morphology was not changed. This indicates that BCA can be well taken up by LLC cells.

### 3.3 Cytotoxicity of LLC cells by BCA-mediated SDT

**FIGURE 3:**
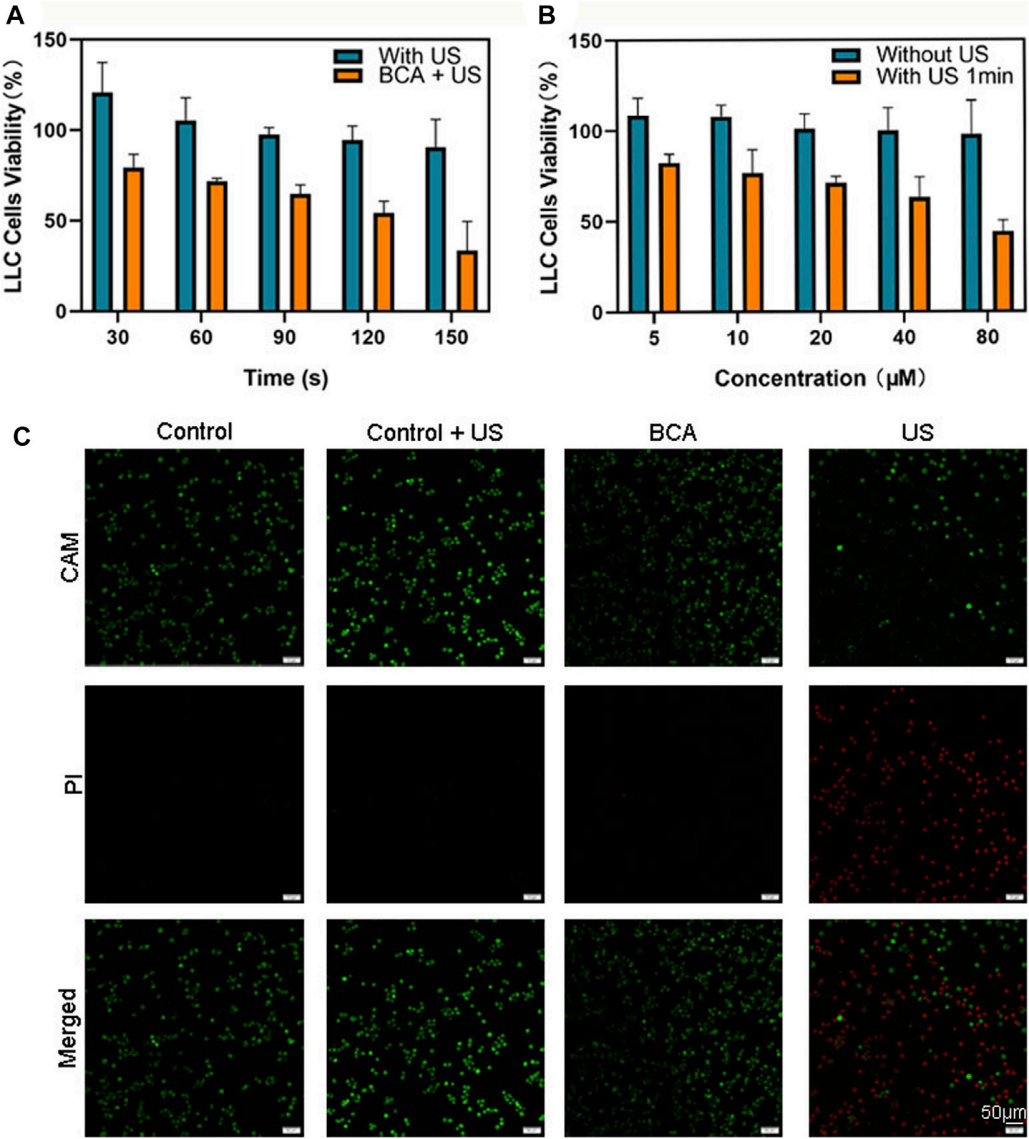
**(A)** Sonodynamic effects, using BCA as a sonosensitizer activated by ultrasound, on the proliferation of LLC cells. **(B)** Effects of ultrasound on the proliferation of LLC cells (data are shown as the mean ± SD of *n* = 3 biologically independent experiments). **(C)** Confocal images of CAM/PI co-stained LLC cells after various treatments. Green fluorescence means live cells, and red fluorescence means dead cells.

Here, we explored the cytotoxicity of BCA and the killing effect of SDT mediated by BCA on lung cancer cells. The treated cells were incubated with BCA for 2 h to evaluate the cytotoxicity of BCA-mediated SDT on LLC cells. Figure 3A illustrates the cytotoxicity of BCA-mediated SDT on LLC cells. Ultrasound only did not cause significant cell death, and when only BCA was combined with ultrasound, there was a significant cytostatic effect, which was time-dependent. When the BCA concentration was fixed at 20 μM, US increased from 30 s to 150 s and cell survival decreased from 79.2% to 33.5%. In addition, BCA-mediated SDT antitumor effect also showed a concentration-dependent manner. When the ultrasound time was set at 1 min, the concentration increased from 5 μM to 80 μM and the cell survival rate decreased from 82.3% to 44%. The toxicity of BCA to LLC cells was analyzed. Figure 3B shows that the cell viability of the BCA only-treated group did not decrease significantly and remained above 94%, indicating high biosafety of BCA. The effects of ultrasound and BCA on the growth of LLC cells were inhibited in a time- and concentration-dependent manner. For the following sonodynamic study, we set the concentration at 20 μΜ because BCA proved safe for both cancer and normal cells at 20 μΜ.

Calcein acetoxymethyl ester (CAM) can stain living cells, with strong green fluorescence (Liminga et al., 1999). The fluorescent dye propidium iodide (PI) is a nuclear staining reagent that can stain DNA. Only dead cells with compromised membrane integrity can be stained by red fluorescent PI (Tawakoli et al., 2013). Therefore, the combination of CAM and PI can simultaneously stain both live and dead cells. The results showed that living cells showed a strong green fluorescence, while dead cells showed a bright red fluoresce. As shown in Figure 3C, most of the cells were dead after BCA-mediated SDT. Only a very small number of dead cells appeared in the BCA only-treated group, and almost no dead cells appeared in the ultrasound only-treated and sham ultrasound-treated groups. These results suggest that BCA-SDT could markedly induce LLC cell apoptosis.

### 3.4 Intracellular reactive oxygen species (ROS)

**FIGURE 4:**
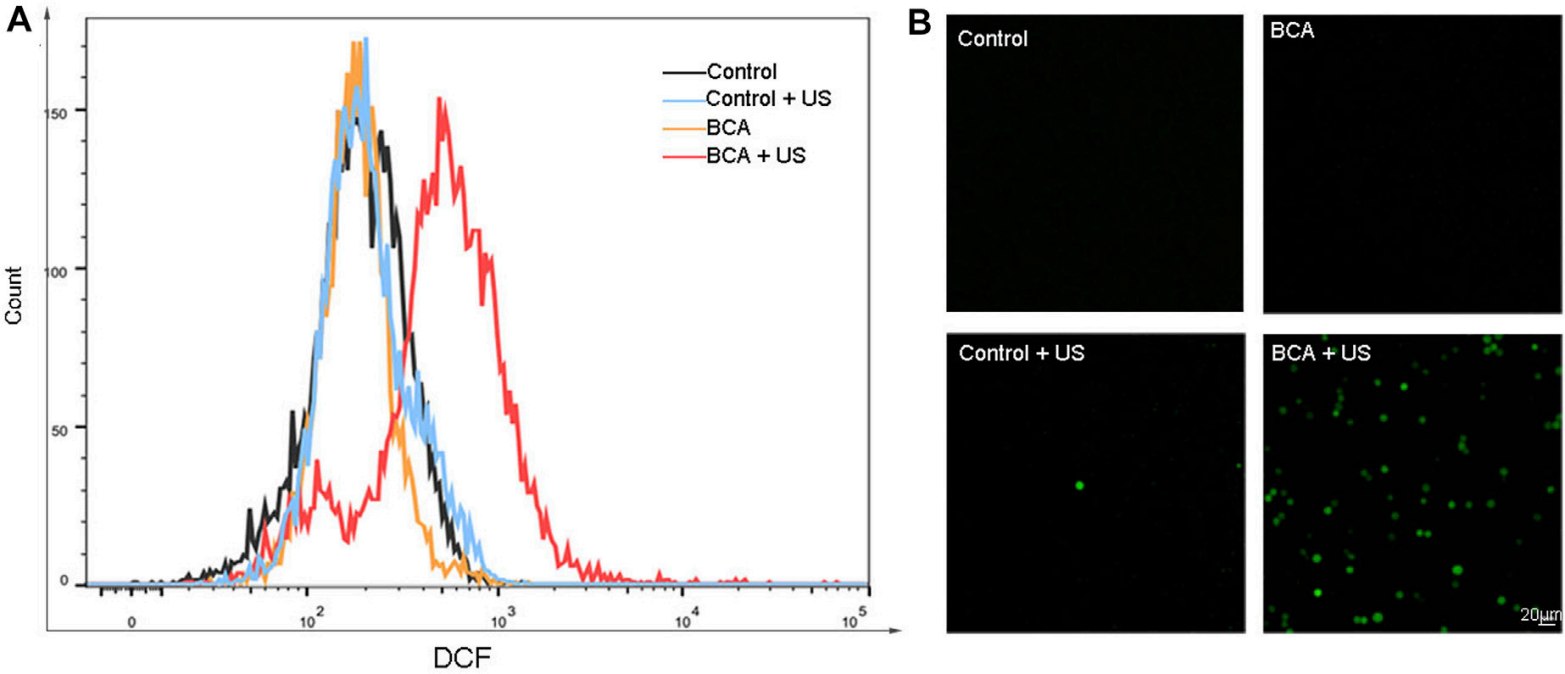
**(A)** Measurement of the ROS level in LLC cells by flow cytometry with DCFH-DA staining. **(B)** Measurement of reactive oxygen species (ROS) in LLC cells, by fluorescence microscopy with DCFH-DA staining 2 h after BCA-mediated sonodynamic therapy at an ultrasonic intensity of 1.5 W/cm^2^ for 90 s; BCA-SDT induces excessive production of intracellular reactive oxygen.

Here, we further explored the mechanism of killing tumor cells by BCA-mediated SDT. The formation of ROS in BCA was quantified by 2,7-dichlorodihydrofluorescein-diacetate (DCFH-DA), which could be converted into strong green fluorescent DCF by cytosolic ROS (Ubezio and Biology, 1994). The fluorescence intensity of ROS in cells was analyzed with DCFH-DA staining by flow cytometry (FCM). Figure 4A shows that the green fluorescence equivalent to the production of ROS is increased only in the BCA combined with ultrasonic activation group, and BCA only or ultrasound only treatment did not increase intracellular ROS levels. This conclusion was supported by fluorescence microscope assay. As shown in Figure 4B, strong green fluorescence was observed 2 h after BCA-mediated SDT compared to other three groups. These results indicate that BCA-mediated SDT has some relationship with ROS formation.

### 3.5 Localization of BCA in LLC cells

**FIGURE 5:**
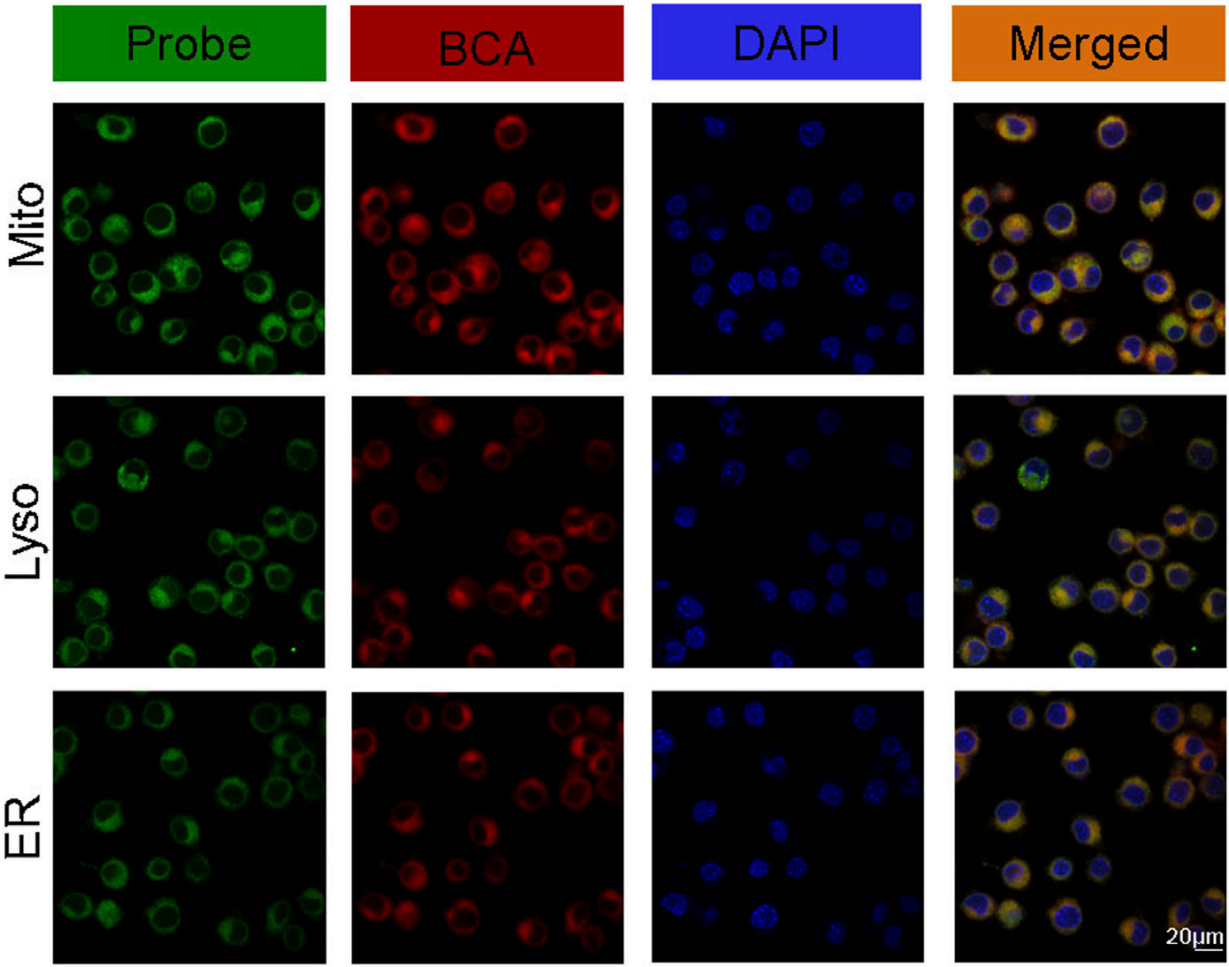
Intracellular localization of BCA in tumor cells after 2-h incubation.

We further studied the localization and targeting of BCA in LLC cells by detecting its inherent fluorescence signal. Mitochondria play an important role in cellular life processes such as energy metabolism, apoptosis regulation, and cell signaling and are thus considered to be the preferred target of SDT. At the same time, the lysosome is also an attractive antitumor target that is associated with the progression of cancer and drug resistance (Tsubone et al., 2017). During SDT, the generated ROS can trigger endoplasmic reticulum stress, which significantly disturbs the growth of cells (Garg et al., 2011; Galluzzi et al., 2012; Gomes-da-Silva et al., 2018). Thus, it is suggested that ultrasound-induced damage to lysosomes, mitochondria, and endoplasmic reticulum may be a promising strategy for the eradication of cancer cells. Based on co-localization experiments, BCA showed stronger signals with yellow or orange fluorescence, indicating that BCA has excellent mitochondrial, lysosomal, and endoplasmic reticulum targeting ability (Figure 5).

### 3.6 Fluorescence imaging effect of BCA *in vitro*

**FIGURE 6:**
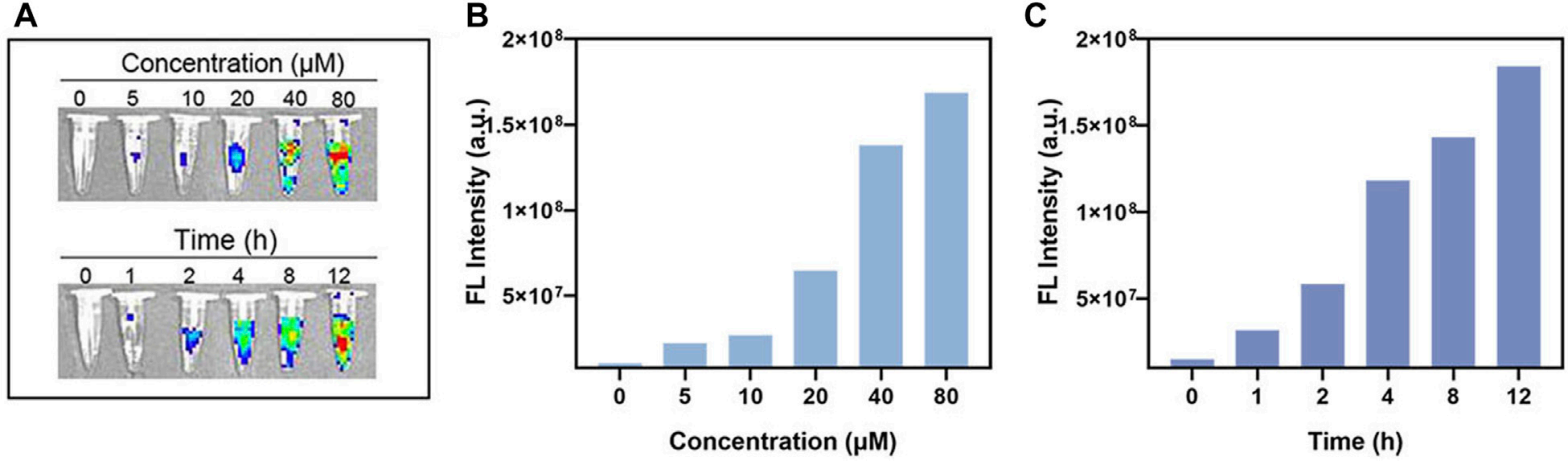
**(A)** *In vitro* FL imaging of BCA. **(B)** *In vitro* FL imaging data statistics of different concentrations of BCA. **(C)** *In vitro* FL imaging data statistics of different times of BCA.

First, we proved that pure BCA solution has good fluorescence imaging function. Here, we further explored its fluorescence imaging function *in vitro*. The NIR-FL (Near Infrared-Fluorescence) imaging of BCA *in vitro* indicated that it has great potential for cell imaging (Figure 6A). Further cellular FL images demonstrated concentration- and time-dependent signal enhancement (Figures 6B, C).

## 4 Conclusion

In this study, we explored, for the first time, BCA combined with near-infrared fluorescence imaging and sonodynamic antitumor. Our study found that BCA can be efficiently internalized by cells and then distributed in mitochondria, lysosomes, and endoplasmic reticulum. Upon ultrasonic activation, BCA can efficiently produce ROS, thereby inhibiting the growth of LLC cells in a time-and concentration-dependent manner. In addition, BCA has good near-infrared fluorescence imaging properties and is expected to be used as a potential diagnostic factor for tumor imaging diagnosis and sonodynamic therapy. However, its effects need to be confirmed in whole animal experiments, and its superiority over other sonosensitizers requires further investigation.

## Data Availability

The original contributions presented in the study are included in the article/Supplementary Material; further inquiries can be directed to the corresponding authors.
